# A Hierarchical Copper Oxide–Germanium Hybrid Film for High Areal Capacity Lithium Ion Batteries

**DOI:** 10.3389/fchem.2019.00869

**Published:** 2020-01-08

**Authors:** Liying Deng, Wangyang Li, Hongnan Li, Weifan Cai, Jingyuan Wang, Hong Zhang, Hongjie Jia, Xinghui Wang, Shuying Cheng

**Affiliations:** ^1^College of Physics and Information Engineering, Institute of Micro-Nano Devices and Solar Cells, Fuzhou University, Fuzhou, China; ^2^NOVITAS, Nanoelectronics Centre of Excellence, School of Electrical and Electronics Engineering, Nanyang Technological University, Singapore, Singapore; ^3^Jiangsu Collaborative Innovation Center of Photovolatic Science and Engineering, Changzhou, China

**Keywords:** self-supported electrode, lithium ion battery, CuO, Ge, areal capacity

## Abstract

Self-supported electrodes represent a novel architecture for better performing lithium ion batteries. However, lower areal capacity restricts their commercial application. Here, we explore a facial strategy to increase the areal capacity without sacrificing the lithium storage performance. A hierarchical CuO–Ge hybrid film electrode will not only provide high areal capacity but also outstanding lithium storage performance for lithium ion battery anode. Benefiting from the favorable structural advance as well as the synergic effect of the Ge film and CuO NWs array, the hybrid electrode exhibits a high areal capacity up to 3.81 mA h cm^−2^, good cycling stability (a capacity retention of 90.5% after 150 cycles), and superior rate performance (77.4% capacity remains even when the current density increased to 10 times higher).

## Introduction

Rechargeable lithium ion batteries (LIBs) are identified as the ideal sources of power for wide applications ranging from portable electronic devices to large-scale products on account of their long-life span and high energy density (Liu et al., 2018b; Xu et al., 2018; Zhang et al., 2018; Yan et al., 2019). However, the specific capacity of the electrodes severely restricts their energy density. As a solution to this problem, different anode materials with higher specific capacities have been investigated to take the place of the present commercial graphite (Kim et al., 2017). To date, Ge and CuO have aroused increasing interest as the novel anodes for new generation LIBs due to their high theoretical capacities. Compared with commercial graphite (theoretical gravimetric capacity is 372 mA h g^−1^), Ge has a high theoretical capacity of 1,624 mA h g^−1^, while CuO has a capacity of 674 mA h g^−1^. Ge has been widely studied because of its high ionic conductivity and low lithiation potential and CuO, as one of the transition metal oxides, has demonstrated to be a promising material for the substitute anodes in LIBs for its earth abundance, commercial benefit, and environmental friendly (Chan et al., 2008; Xiaojun et al., 2012; Huang et al., 2015; Susantyoko et al., 2015; Mironovich et al., 2017).

However, the considerable capacities of Ge and CuO are generally accompanied by drastic volume change upon Li intercalation and deintercalation, thus causing the poor cycling performance (Liu et al., 2015; So et al., 2018). Great efforts have been made to solve the pulverization problem during the Li insertion and extraction process using nanomaterials such as nanoparticles (Hyojin et al., 2005; Mi-Hee et al., 2010; Yang et al., 2010), nanowires (Chan et al., 2008; Chockla et al., 2012; Yuan et al., 2012; Mullane et al., 2013), nanotubes (Chen et al., 2009; Cao et al., 2015; Liu et al., 2018a; Sun et al., 2018), and so on. For example, Li et al. synthesized mesoporous and hollow Ge@C nanostructures via carbon coating and reduced the hollow ellipsoidal GeO_2_ precursor into Ge. A stable cycling performance (capacity retention remained 100% at 0.2 C rate for 200 cycles) and high rate capability (805 mA h g^−1^ at 20°C) is finally obtained (Li et al., 2013). Wang et al. fabricated self-supported CuO nanowires (NWs) on Cu foam (CF). The obtained electrodes delivered a specific capacity of 461.5 mA h g^−1^ after 100 cycles at a current density of 100 mA g^−1^, and a capacity of 150.6 mA h g^−1^ even at a high rate of 1,000 mA g^−1^ (Wang et al., 2018).

Although these nanoengineering strategies have effectively improved the Li^+^ ions storage performance of these high-capacity electrodes (Sun et al., 2018), most of these nanoscale metal oxides/group-IV elements and corresponding composites are mixed with organic binders and conductive carbon and then fabricate into electrode, which complicate the fabrication process (Wang et al., 2012). The bonding force between traditionally used binders and high-capacity active materials is too weak to maintain a stable performance after long-term cycling (Chang et al., 2019).

Recently, self-supported active nanomaterials which is *in situ* grown on current collectors without any inactive materials represent an unique architecture, which can offer many advantages for LIBs such as large contact area with electrolyte, great electrical conductivity, fast Li-ion transportation, and better performance for the electrodes (Wang et al., 2016). Susantyoko et al. fabricated amorphous Ge on the multiwall carbon nanotube arrays (MWCNT/a-Ge) by the combination of facial chemical vapor deposition and a physical sputtering method, which could give a specific areal capacity of 0.405 mA h cm^−2^ at the rate of 0.1 C after 100 cycles (Susantyoko et al., 2014). Kim et al. synthesized nano-Ge/C composite via electrochemical deposition method; the obtained electrode exhibits a capacity of 1 mA h cm^−2^ at 0.1 C over 50 cycles (Kim et al., 2017). Ji et al. fabricated binder-free electrodes, which is composed of 3D graphene network and octahedral CuO, the obtained 3D GN/CuO composites, yielding an areal capacity of 0.39 mA h cm^−2^ at 0.095 mA cm^−2^ (Dong et al., 2016). Xu et al. synthesized CuO mesocrystal entangled with MWCNT composites through a combination of precipitation and an oriented aggregation process. The CuO-MWCNT composites could deliver an areal capacity of 1.11 mA h cm^−2^ after 400 cycles at the current density of 0.39 mA cm^−2^ (Xu et al., 2016). Great progress for CuO- and Ge-based self-supporting electrodes has been achieved by the above-mentioned effects. Areal capacity is one of the important parameters for practical LIB application. Especially for self-supporting electrodes, it is very important and hard to obtain both high areal capacity and good electrochemical performance. However, the areal capacities of the most obtained electrodes are <2 mA h cm^−2^, which is lower than the commercial specification of 3–4 mA h cm^−2^ (Cong et al., 2017). Normally, larger mass loading of active materials will make contribution to higher areal capacities but meanwhile sacrificing electrochemical performance (Chang et al., 2019). There is an increasing concern about fabricating self-supporting electrodes with high areal capacity as well as good electrochemical performance.

**Figure 1:**
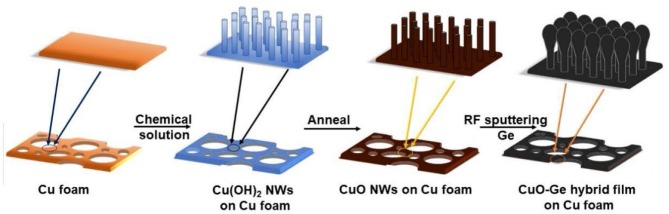
Schematic illustration of the fabrication processes of the CuO–Ge hybrid film on Cu foam (CF).

Usually, the self-supporting electrodes cannot maintain good electrochemical performance at very high areal capacity. Herein, we report a hierarchical CuO–Ge hybrid film on CF as a self-supporting electrode with ultrahigh areal capacity for LIB application. As shown in Figure 1, the integrated film was formed by physical vapor deposition of Ge film on CuO NWs array, which were grown directly on the CF via a facial and scalable solution approach. CuO NWs array with well-defined nanostructure serves as both the active materials and conductive connection for Ge film. The porous feature will not only alleviate the drastic volume change during the Li insertion and extraction process but also facilitate the diffusion of electrolyte into the electrode. Benefiting from the favorable nanostructures as well as the synergic effect of the Ge film and CuO NWs array, the integrated electrode delivers ultrahigh areal capacity, extraordinary rate capability, and stable cycling performance. Moreover, this is the first time that CuO NWs combined with Ge film hybrid anode achieved a high areal capacity. It could deliver an ultrahigh charge areal capacity up to 3.45 mA h cm^−2^ after 150 cycles at a current density of 0.8 mA cm^−2^ and a capacity ~2.98 mA h cm^−2^ at a current density as high as 4 mA cm^−2^.

## Experimental

### Fabrication Procedure

#### Fabrication of CuO NWs Array

Typically, the growth of CuO NWs array on CF is fabricated from a simple and scalable method. The CF with dimension of 2 × 2 cm^2^ (1 mm, 100 PPI) was first degreased in 1.0 mol L^−1^ HCl for 15 min and then washed in ethanol, acetone, and deionized water by ultrasonication for 15 min, respectively. The rinsed CF was then submerged in the mixed solution, which was prepared by adding 8 ml of freshly obtained 10 M NaOH into (NH_4_)_2_S_2_O_8_ solution [0.913 g (NH_4_)_2_S_2_O_8_ added into 22 ml of deionized water] with magnetically stirring. After 15 min reaction time, the light blue CF can be obtained, which means the formation of Cu(OH)_2_ NWs. After rinsing with deionized water for several times, the light blue CF was dried under N_2_ gas flow and annealed at 180°C in air at a ramp rate of 2°C min^−1^ for 2 h. Then, the final product CuO NWs array was obtained with dark brown color. The principal of CuO NWs array *in situ* grown on CF could be described as following reactions (Cheng et al., 2016):

(1) $$\begin{array}{l} \text{Cu}+4{\text{OH}}^{-}+{\left({\text{NH}}_{4}\right)}_{2}{\text{S}}_{2}{\text{O}}_{8}\to \text{Cu}{\left(\text{OH}\right)}_{2}+2{\text{SO}}_{4}^{2-}\\ \;+2{\text{NH}}_{3}↑+2{\text{H}}_{2}\text{O} \end{array}$$

(2) $$\begin{array}{l} {\text{Cu}\left(\text{OH}\right)}_{2}\to \text{CuO}+{\text{H}}_{2}\text{O} \end{array}$$

#### Fabrication of Hierarchical CuO–Ge Hybrid Film

The CuO NWs array on CF was placed into an radio frequency (RF) sputtering system (Verios G4 UC, Shenyang Lining Co.) using 99.999% pure Ge target. The base pressure was 7.8 × 10^−4^ Pa. Then, argon flowed at 50 sccm, and pressure remained at 2.2 Pa. The RF power was 100 W, and the deposition time was 400 min. The mass loading of CuO NWs supported Ge (typically ~0.67 mg cm^−2^), CuO NWs array (typically ~3.64 mg cm^−2^) and Ge on pristine CF (typically ~0.45 mg cm^−2^) were weighed before and after sputtering using a microbalance (OHAUS, AX224ZH) with an accuracy of 0.1 mg. Besides, the Ge film was also deposited on pristine CF under the same deposition parameters.

### Structural Characterization

The samples were characterized using X-ray diffraction (Rigaku Ultima IV) and Raman spectroscopy (WITEC alpha300 R Confocal Raman system), and the structure and morphology characterization of them were carried out by field-emission scanning electron microscopy (SEM, FEI Inspect F50) with accelerating voltage of 5.00 kV and transmission electron microscopy (TEM, FEI Tecnai G2).

### Electrochemical Characterization

CR 2032-type coin cells was used to test electrochemical characterizations, test cells were assembled in a high-purity argon filled glove box (Mikrouna Technology) with oxygen and moisture level <0.5 ppm. The fabricated self-supporting electrodes were used as the working electrode and a Li foil as the counter and reference electrode. Lithium hexafluorophosphate (LiPF_6_) (1 M) in a mixture of ethylene carbonate and diethyl carbonate (1:1 in volume) was used as the electrolyte. All the cells were aged for 12 h so that the electrolyte can fully infiltrate the whole cell before measurement. Lithium storage performance were evaluated by a multichannel battery tester (Neware, BTS-610) in the voltage range of 3.0–0.01 V (Li/Li^+^). An electrochemical workstation (CHI660c, Shanghai Chenhua Co.) was used to evaluate the cyclic voltammetry (CV) at scan rate of 0.1 mV s^−1^. All the tests were carried out in the thermotank at fixed temperature of 25°C.

## Results and Discussion

**Figure 2:**
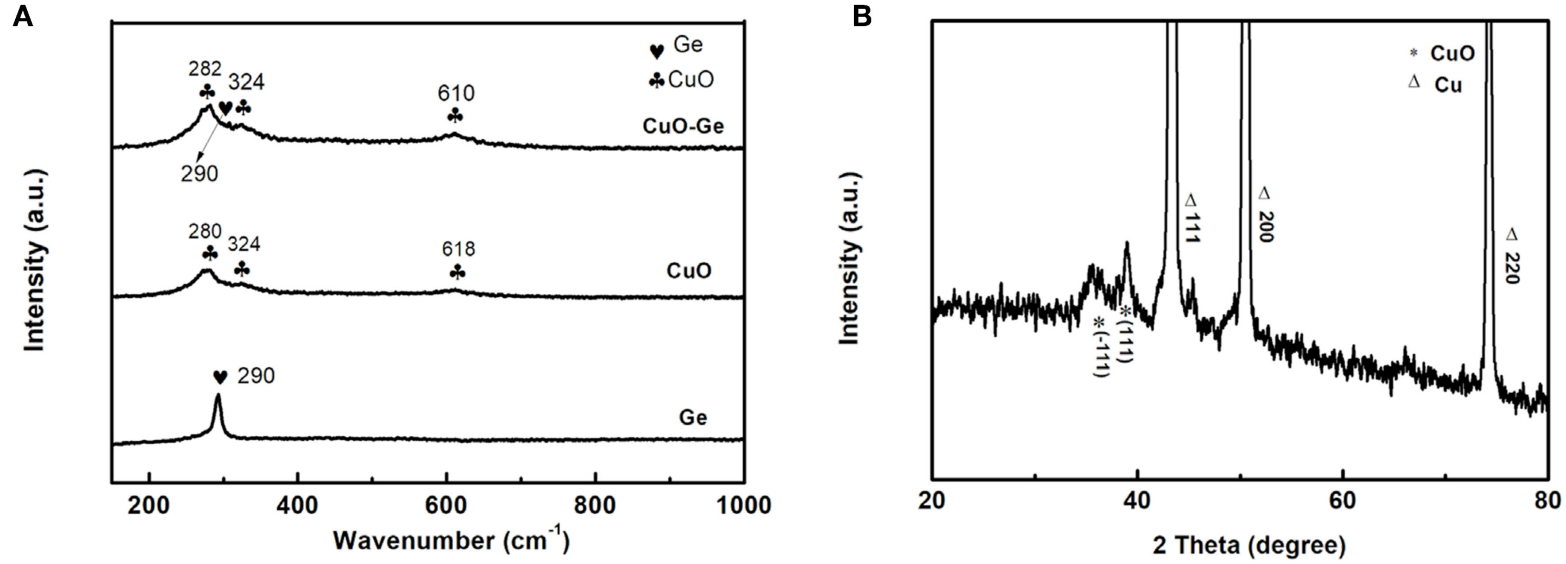
**(A)** Raman spectrum of Ge, CuO NWs array, and CuO–Ge hybrid film; **(B)** X-ray diffraction (XRD) pattern of the CuO–Ge hybrid film.

The Raman spectra analyses of the Ge film, CuO NWs array, and CuO–Ge hybrid film are shown in Figure 2A. For Ge film, a broad peak at 290 cm^−1^ was observed, which can be indexed to amorphous form of Ge (Susantyoko et al., 2014). There are three Raman peaks at 280, 324, and 618 cm^−1^ for CuO NWs array samples, which can be indexed to the A_g_, ${\text{B}}_{\text{g}}^{\left(1\right)}$, and ${\text{B}}_{\text{g}}^{\left(2\right)}$ modes of CuO (Xu et al., 2015). All the peaks can be found on the CuO–Ge hybrid film samples, demonstrating that the hybrid structure was successfully fabricated. The X-ray diffraction patterns are shown in Figure 2B. One can note that the strong diffraction peaks of 43.3, 50.4, and 74.1°, which could be indexed to the CF with JCPDS card no. 70-3039. There are two weak but identifiable peaks located at 35.5 and 38.8°, corresponding to the (−111) and (111) planes of the monoclinic CuO, with JCPDS card no. 89-5899.

**Figure 3:**
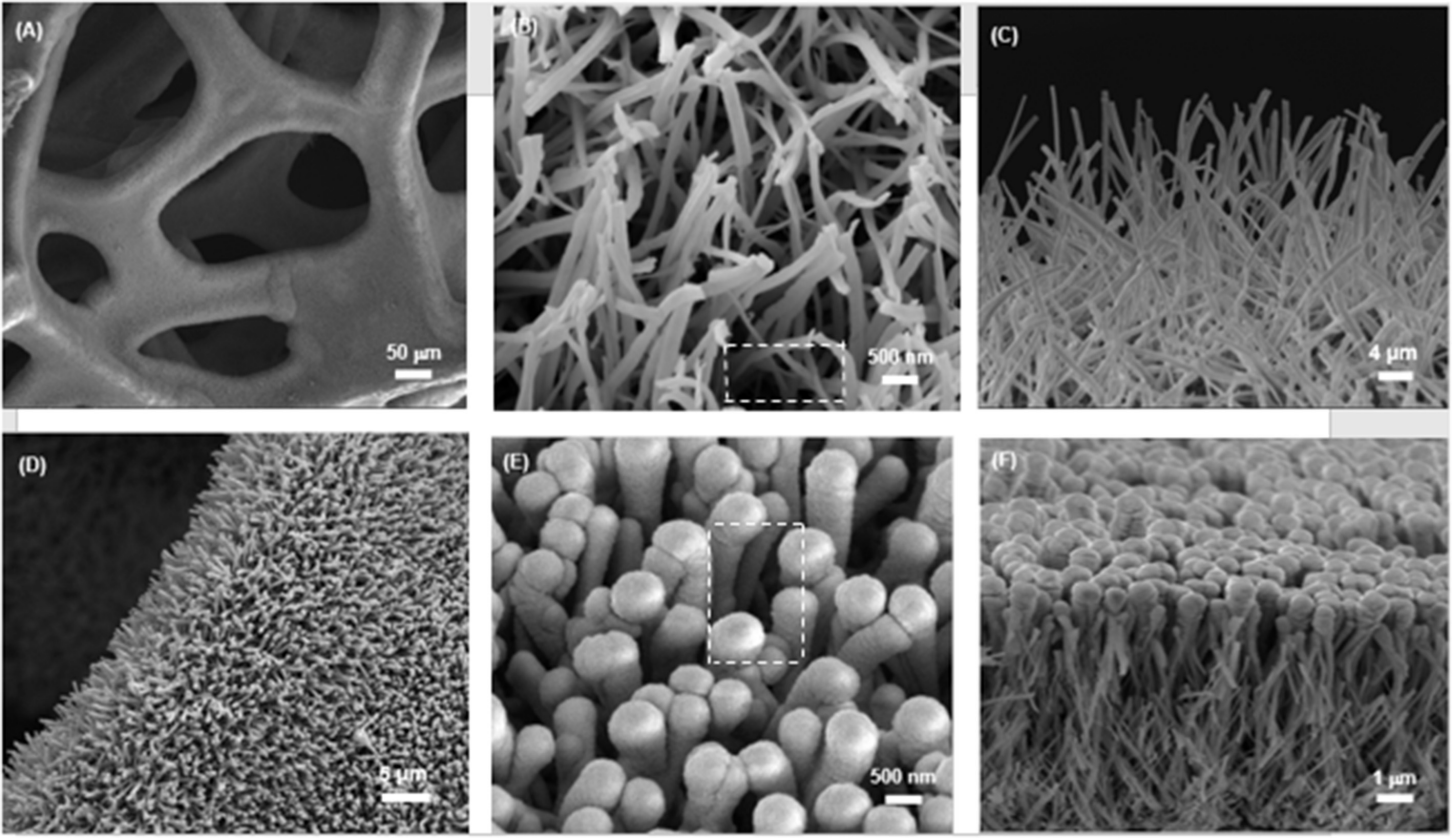
**(A,B)** The top and **(C)** side view SEM images of CuO NWs array; **(D,E)** The top and **(F)** side view SEM images of the CuO–Ge hybrid film.

The typical SEM images of the obtained CuO NWs array on CF are shown in Figures 3A–C. The low-magnification SEM image in Figure 3A shows that CF has a well-organized 3D porous architecture. The magnified image shown in Figure 3B indicates the aligned CuO NWs array are 192 nm in diameter, and there are sufficient space available in CuO NWs array as indicated by the white-dashed squares, which can provide room for Ge thin film loading. Besides, the side view SEM image shown in Figure 3C demonstrates that all the CuO NWs array with length of ~6.1 μm are firmly rooted from Cu microfibers.

Then, a thick amorphous Ge was sputtered on the CuO NWs array using RF sputtering technique. Figures 3D–F show the SEM images of the CuO–Ge hybrid film. A typical image of a part of CF, as shown in Figure 3D, indicates that the 3D ordered nanowire architecture are well preserved after Ge coating. From Figures 3E,F, it is clearly observed that the average diameters and length of the nanowires are increased to 587.5 nm and 6.6 μm, respectively, due to the deposition of Ge film. There are still large space in between these nanowires after sputtering as indicated by the white-dashed squares in Figure 3E, which will not only benefit for accommodating the volume change but also facilitate the diffusion of electrolyte into the electrode. From the side view SEM images shown in Figure 3F, it can be seen that the diameter of the synthetic nanowires is gradually decreased from the top to the bottom, which is attributed to the shadowing effect of the RF sputtering technique. It has been demonstrated that this structure is beneficial for Li storage performance (Wang et al., 2016).

**Figure 4:**
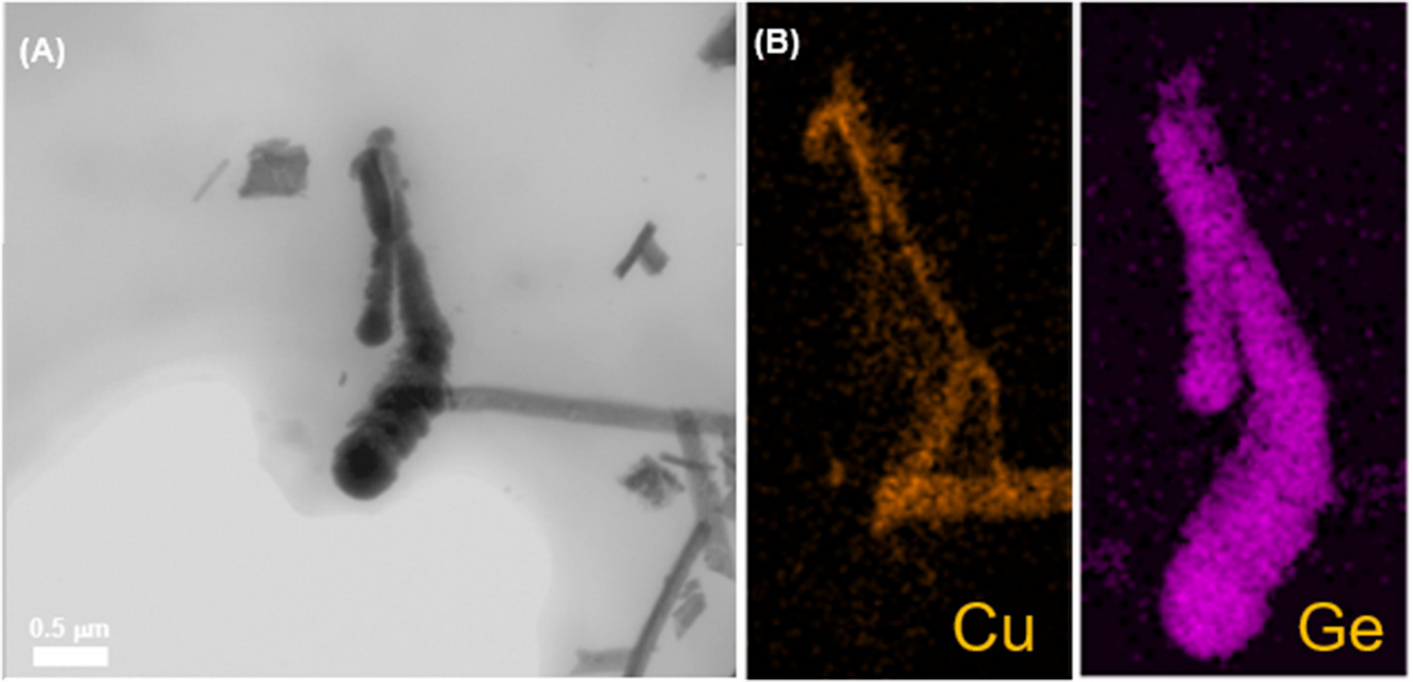
**(A)** Annual dark-field TEM image of the CuO–Ge hybrid film; **(B)** elemental mapping of CuO–Ge hybrid film: the corresponding Cu, Ge EDX maps.

We also present the TEM images of the CuO–Ge hybrid film in Figure 4A. The obtained distributions of Cu and Ge are shown in Figure 4B, the energy dispersive spectroscopy mapping profile obviously pictures that the outer sheath consists of Ge, whereas Cu is perfectly populated in the inner part of the CuO–Ge hybrid film, and the Ge films were grown uniformly and was deposited onto the whole CuO NWs.

**Figure 5:**
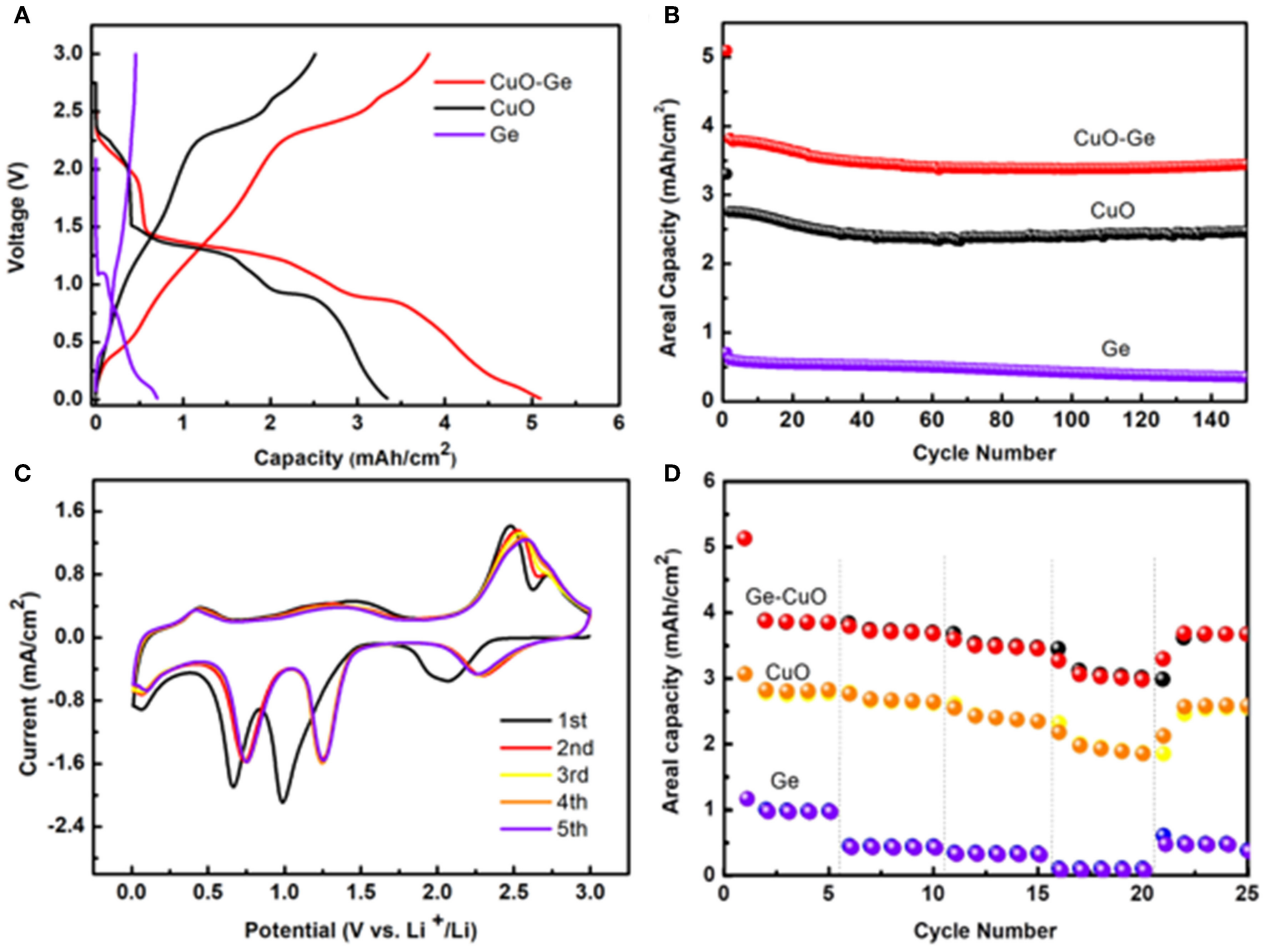
**(A)** The initial voltage profiles of the CuO–Ge hybrid film, CuO NWs array, and Ge film; **(B)** the cycle performance of the CuO–Ge hybrid film, CuO NWs array, and Ge film; **(C)** the initial five cyclic voltammetry (CV) curves of the CuO–Ge hybrid film; **(D)** rate performance of CuO–Ge hybrid film, CuO NWs array, and Ge film.

Figure 5A shows the typical initial discharge and charge profiles of the CuO–Ge hybrid film, CuO NWs array, and Ge film with the voltage window of 0.01–3 V (Li^+^/Li) at a current density of 0.8 mA cm^−2^. The CuO–Ge hybrid film delivers an initial discharge and charge capacity of ~5.09 and 3.81 mA h cm^−2^, giving the initial Coulombic efficiency of 74.8%. The irreversible discharge capacity is associated with the formation of solid electrolyte interface layer and the irreversible insertion of Li^+^ into CuO and Ge films, which are common for CuO and Ge based anodes (Chan et al., 2008; Chockla et al., 2012; Yuan et al., 2012; Mullane et al., 2013; Liu et al., 2018a). The low Coulombic efficiency may restrict the capacity of anode materials; however, it has been demonstrated that LIBs must undergo a few charge–discharge cycles, which is generally called the “formation process” (Chen et al., 2013). Besides the first cycle, the Coulombic efficiency of the battery was all above 99.2%, indicating excellent recyclability. In contrast, the first discharge and charge capacities are 3.34 and 2.50 mA h cm^−2^ for CuO NWs array and 0.71 and 0.59 mA h cm^−2^ for Ge film, respectively. The CuO–Ge hybrid film electrode exhibits much higher initial charging areal capacity when compared with the sum of the Ge film and CuO NWs array electrode. This is because the mass loading of the Ge film on CuO NWs array is higher than that on CF attribute to the larger surface area of the CuO NWs array, demonstrating the structural advantages of the CuO NWs array.

Figure 5B compares the cycle performance of the CuO–Ge hybrid film, CuO NWs array and Ge film for subsequent 150 cycles at a high current density of 0.8 mA cm^−2^. For the CuO–Ge synthesized film electrode, a reversible discharge capacity of 3.81 mA h cm^−2^ was achieved at the second cycle, corresponding to a specific capacity of 883 mA h g^−1^. The electrode could still deliver a high areal capacity of ~3.45 mA h cm^−2^ with a capacity retention of 90.5% after 150 cycles (the corresponding specific capacity contribution from Ge was 1,462 mA h g^−1^, and CuO was 678 mA h g^−1^). In contrast, the CuO NWs array electrode could deliver a capacity of 2.47 mA h cm^−2^ after 150 cycles, which corresponds to the 90.0% of the original one. While Ge film can only obtain a reversible capacity of 0.2 mA h cm^−2^ after 150 cycles with a much lower capacity retention of 59.3%. The CuO–Ge hybrid film electrode exhibits a superior improvement in Li storage performance compared to the other electrodes, which may be attribute to the novel structure design using a hierarchical 3D nanostructure to combine two high theoretical capacity materials. The well-separated CuO NW arrays will not only provide large area for larger mass loading of Ge but also the large void space to overcome the large volume change during charge and discharge.

The initial few CV curves of the CuO–Ge hybrid film electrode were conducted in range of 0.01–3.0 V at a scan rate of 0.1 mV s^−1^ as displayed in Figure 5C. It can be indicated from these peaks that there is a multistep electrochemical reaction between Li and the hybrid electrode. A broad but moderate peak at ~2.1 V corresponding to the initial formation of Li_*x*_CuO in the first cathodic sweep. Then, two succession reduction peaks were observed at ~1.0 and ~0.66 V, corresponding to the transformation of Li_x_CuO into Cu_2_O and Cu; then, a moderate peak at ~0.07 V was observed, which was associated with the formation of the Li_x_Ge alloy (Rudawski et al., 2013; Guo et al., 2015). These reduction peaks shifted toward slightly higher voltages in the following scans, which might associate with the drastic Li driven structural modifications during the initial discharge and charge process (Yunhua et al., 2014). A peak was found at 0.44 V during the first anodic scan, attributing to the phase transition of Li_*x*_Ge to Ge; then, two distinct peaks at ~1.5 and ~2.5 V and a shoulder peak at ~2.7 V appeared, which was associated with oxidation of Cu^0^ to Cu^+^ and Cu^2+^ (Dong et al., 2016; Xu et al., 2016; Wang et al., 2018). These results are in agreement with the other reports of electrochemical reactions of Ge and CuO with Li (Seo et al., 2011; Ren et al., 2013; Xinghui et al., 2014; Wei et al., 2017; Lin et al., 2018; Wang et al., 2018). The CV curves were well-overlapped with each other from the second cycle afterwards, suggesting that the electrode has a good reversibility.

The rate capability is further tested for the CuO–Ge hybrid film electrode, which is of significant importance for high power energy storage. The rate performance was evaluated by charging–discharging at varied current densities varying from 0.4 to 4 mA cm^−2^. As shown in Figure 5D, after the first five cycles at the current density of 0.4 mA cm^−2^, the obtained electrode showed a high discharge areal capacity of 3.85 m A h cm^−2^; then, it slightly reduced to 3.68 and 3.45 m A h cm^−2^ at current rates of 0.8 and 1.6 mA cm^−2^. Even at a rate as high as 4 mA cm^−2^, the CuO–Ge hybrid film could still deliver a reversible capacity of ~2.98 m A h cm^−2^, corresponding to the 77.4% capacity of the capacity at 0.4 mA cm^−2^. After the rate returned back to the initial value of 0.4 mA cm^−2^ for five cycles, 94.5% of the initial charge capacity was regained. In the comparison, the CuO NWs exhibited a capacity of 2.83, 2.65, 2.35, and 1.86 m A h cm^−2^, respectively, and eventually obtained a capacity of 2.59 m A h cm^−2^. As for the Ge film, it only showed a capacity of 0.98, 0.44, 0.33, and 0.10 m A h cm^−2^, respectively. Indicating the benefit from the favorable nanostructures as well as the synergic effect of the Ge film and CuO NWs array, the hybrid electrode hybrid film electrode has wonderful rate capability far beyond the CuO NWs and Ge film electrode.

**Figure 6:**
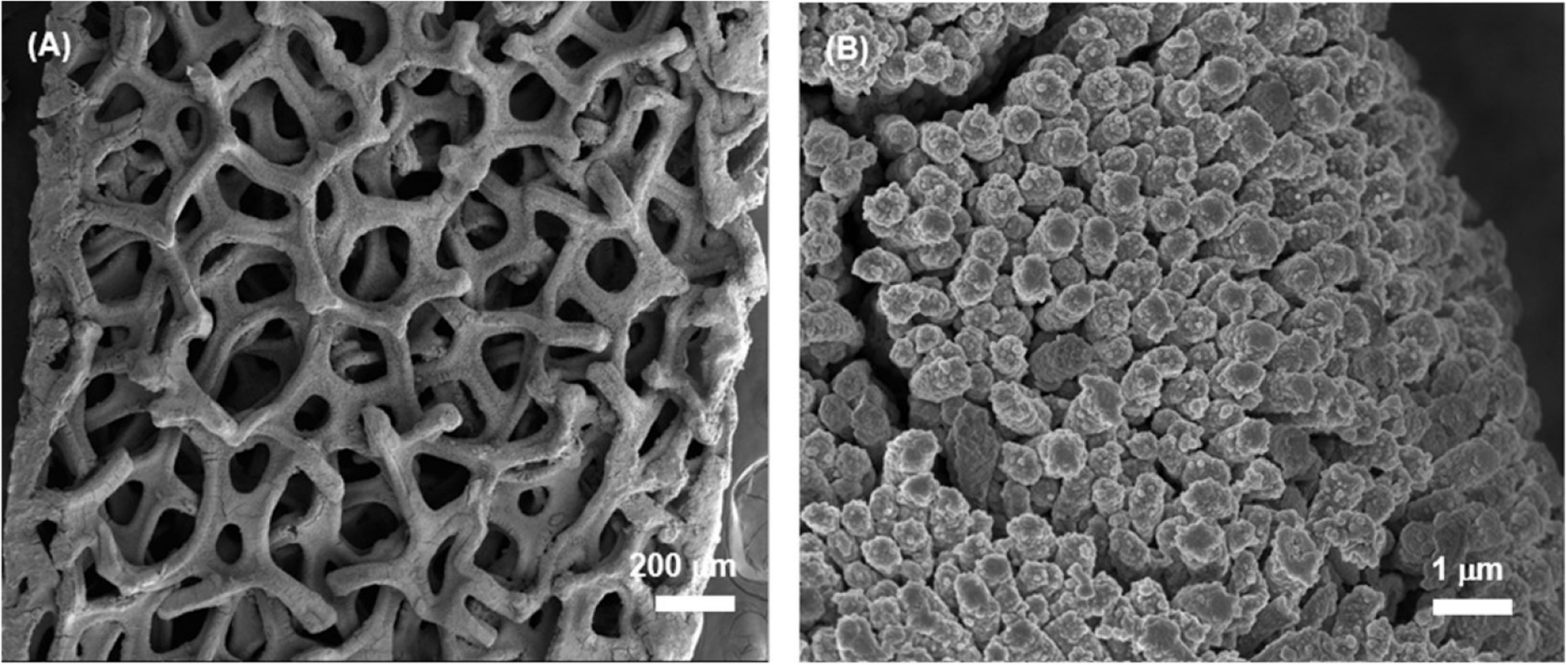
**(A,B)** SEM images of the CuO–Ge hybrid film after 50 cycles.

To examine the structure stability of the CuO–Ge hybrid film electrode upon repeated discharge/charge process, the electrode was disassembled after 50 cycles. As shown in Figure 6A, the CuO–Ge hybrid film were uniformly remained on the CF with no detaching signs. From high magnification SEM shown in Figure 6B, one can see that the hierarchical CuO–Ge hybrid films maintain their original structure even after 50 cycles, indicating high structural stability of the hybrid structure, which proves that this novel structure can withstand the dramatic volume change caused during repeated discharge and charge cycles. Therefore, the excellent lithium storage performance of the CuO–Ge hybrid film electrode is mainly due to the following aspects: (1) The well-separated CuO NW arrays can not only provide large area for higher mass loading of Ge, resulting in higher areal capacity, but also improve the cycle performance of the Ge film by providing sufficient void space to alleviate the large volume change of the Ge film. (2) The hierarchical porous feature in the hybrid film not only provides sufficient space to accommodate the drastic volume change but also facilitates the lithium diffusion into the inner electrodes. (3) The Cu in the lithiated CuO NWs will promote the electronic conductivities, enhancing the rate performance of the electrodes (Yang et al., 2014).

## Conclusions

In conclusion, an efficient strategy to prepare self-supporting electrode with ultrahigh areal capacity for LIB application has been introduced. The obtained CuO–Ge hybrid film electrode exhibits excellent lithium storage performance. It can deliver a high areal capacity of 3.81 mA h cm^−2^ after 150 cycles, corresponding to 90.5% of the original one. Furthermore, the electrode could deliver high areal capacities of 2.98 mA h cm^−2^ even at ultrahigh current density of 4 mA cm^−2^. The hierarchical CuO–Ge hybrid film grown directly on CF could be a novel substitute of graphite for LIBs, and the facial and efficiency synthesis strategy sheds light on improving the areal capacity of the self-supporting electrodes, which can be applicable for preparation of other high capacity hybrid electrode for energy storage application.

## Data Availability Statement

All datasets generated for this study are included in the article/supplementary material.

## Author Contributions

All authors have contributed in various degrees to the analytical methods used, to the research concept, to the experiment design, to the acquisition of data, or analysis and interpretation of data, to draft the manuscript, or to revise it critically for important intellectual content.

### Conflict of Interest

The authors declare that the research was conducted in the absence of any commercial or financial relationships that could be construed as a potential conflict of interest.
